# Prognostic Value of Tumor Mutational Burden Related to Immune Infiltration in Cervical Squamous Cell Carcinoma

**DOI:** 10.3389/fmed.2021.755657

**Published:** 2021-11-11

**Authors:** Fang Wen, Shuai Ruan, Wenjie Huang, Xiaoxue Chen, Yulan Wang, Suping Gu, Jiatong Liu, Shenlin Liu, Peng Shu

**Affiliations:** ^1^Department of Oncology, Affiliated Hospital of Nanjing University of Chinese Medicine, Nanjing, China; ^2^Department of Oncology, Jiangsu Province Hospital of Chinese Medicine, Nanjing, China; ^3^First Clinical Medical College, Nanjing University of Chinese Medicine, Nanjing, China

**Keywords:** tumor mutation burden, immune infiltration, prognosis, cervical squamous cell carcinoma, TCGA

## Abstract

Cervical squamous cell carcinoma is one of the most common causes of female cancer deaths worldwide. At present, immunotherapy using immune checkpoint blockade (ICB) has improved the prognosis of many cancer patients, and neoantigens generated by mutations may serve as potential biomarkers for predicting the outcome of ICB therapy. In this study, we identified missense mutations as the most frequent in landscapes of gene mutation in cervical squamous cell carcinoma (CESC) samples. Patients with higher tumor mutation burden (TMB) presented higher overall survival (OS). In addition, there was a significant correlation between the high TMB group and fractions of most immune cells. Univariate and multivariate Cox regression analyses identified five hub genes (IFNG, SERPINA3, CCL4L2, TNFSF15, and IL1R1) that were used to build a prognostic model. In the prognostic model, the low-risk group achieved better OS. Mutations in the five hub genes mainly affected the infiltration level of CD8+ T cells and dendritic cells. In conclusion, our study is valuable for exploring the role of TMB and its relationship with immune infiltration in CESC. Moreover, the prognosis model may help predict the sensitivity of patients to immunotherapy and provide underlying biomarkers for personalized immunotherapy.

## Introduction

Cervical squamous cell carcinoma (CESC) is a female cancer with high morbidity and mortality. Although studies have shown that cervical squamous cell carcinoma (CESC) can occur in women of different ages, the average age at diagnosis is lower than that of most cancers (1). Hysterectomy and human papillomavirus (HPV) screening can reduce the mortality of patients with CESC, but the 5-year survival rate is still not satisfactory (2). Thus, it is imperative to develop new treatments.

Immune checkpoint blockade immunotherapy can effectively induce anti-tumor immunity of different solid tumors, although few patients benefit from the treatment. Tumor cells damage the immune system of the body through a variety of suppression mechanisms, such as activation of immune regulatory checkpoints (3). Immune checkpoint blockade (ICB) treatment can reactivate the cytolytic potential of cytotoxic T cells and increase the effective elimination of tumor cells by interrupting dysfunctional “self-tolerance” signaling. Moreover, various types of tumors have widely different response rates to ICB; and in most solid tumor types, ~25% of patients are sensitive to ICB therapy (4). Riaz et al. demonstrated that insufficient expression of mismatch repair (MMR) mechanisms increased the number of tumor cells presenting encoded and mutation-associated neoantigens, which can be recognized by the immune system of the body (5). Identifying a population of patients who are more likely to benefit from new antibodies produced by non-synonymous mutations is becoming increasingly important in clinical research.

Tumor mutation burden is an emerging biomarker that predicts the response of patients with tumor to ICB immunotherapy. At present, surrogate biomarkers of tumor mutation burden (TMB) have been developed and found to be associated with the clinical efficacy of ICB therapy (6, 7). Accurate evaluation of the TMB status may play a key role in the successful outcome of immunotherapy with ICB (8), as the status of gene mutation is closely related to overall survival (OS). Based on the results of a survival analysis, Park et al. observed that breast cancer patients with high TMB [human epidermal growth factor receptor 2 (HER2) positive] had longer survival (9). Similarly, Goodman et al. demonstrated that patients with melanoma and non-small cell lung cancer and high TMB had better progression-free survival (PFS) than those with low TMB (10). Taken together, TMB-based immunotherapy is a promising individualized approach for patients with cancer.

Genomic instability caused by long-term HPV infection can lead to gene mutations in cells, and these mutated genes can crosstalk with tumor suppressor pathways, which results in CESC transformation and malignant progression (11). Furthermore, HPV infection significantly affects the expression of programmed death ligand 1 (PD-L1) in CESC tissues (12). A number of studies have confirmed that HPV-positive status is positively correlated with increased PD-L1 expression (13, 14). In this study, we investigated the relationship between TMB and OS rate, and clarified the association between TMB and clinical parameters. Furthermore, we evaluated the prognostic effects of TMB on CESC and its underlying correlation with immune cell infiltration by constructing a prognostic model related to TMB.

## Results

### The Landscape of Somatic Mutations in CESC

**Figure 1:**
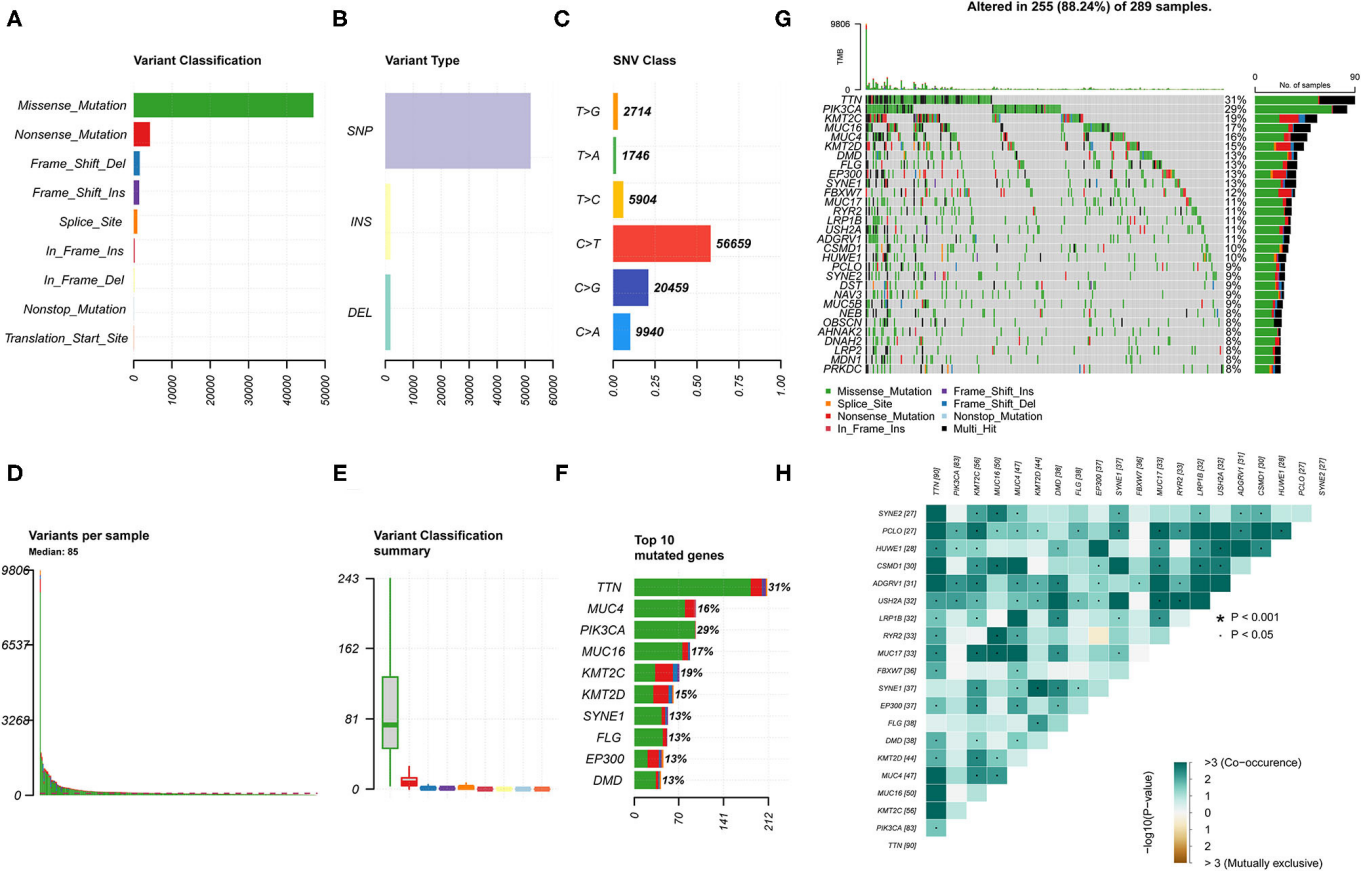
Landscape of gene mutation in the cervical squamous cell carcinoma (CESC) samples. **(A,B)** Variant classification and variant types identified in CESC. **(C)** Single nucleotide variant (SNV) classes in CESC. **(D)** Number of variations in different samples. **(E)** Summary of the median value of variant classification. **(F)** Top 10 mutated genes in CESC. **(G)** Waterfall plot showing mutation information of frequently mutated genes in each sample. The bar plot above represents the tumor mutation burden. **(H)** Co-occurrence and mutually exclusive among mutated genes.

A total of 307 CESC samples were downloaded from the Cancer Genome Atlas (TCGA) database. We used the R package *maftools* to visualize mutation data results. Comprehensive statistics of the gene mutation data of the CESC samples indicated missense mutations were the most frequent (Figure 1A); single nucleotide polymorphism (SNP) was the most common type of mutation (Figure 1B); C > T was the main single nucleotide variant (SNV) (Figure 1C). In addition, the number of mutations in each sample and the median value of different mutation types were classified (Figures 1D,E). The top 10 mutant genes of CESC were TTN (31%), PIK3CA (29%), KMT2C (19%), MUC16 (17%), MUC4 (16%), KMT2D (15%), SYNE1 (13%), FLG (13%), EP300 (13%), and DMD (13%) (Figures 1F,G). In addition, the correlation diagram revealed that a co-occurrence relationship among these mutant genes was dominant (Figure 1H). Specific details associated with the mutation status are shown in Supplementary Figure 1. The clinical data of patients with CESC used for the subsequent survival analysis and related clinical parameter analysis are presented in Supplementary Table 1.

### Correlation of TMB With Prognosis and Clinical Parameters

**Figure 2:**
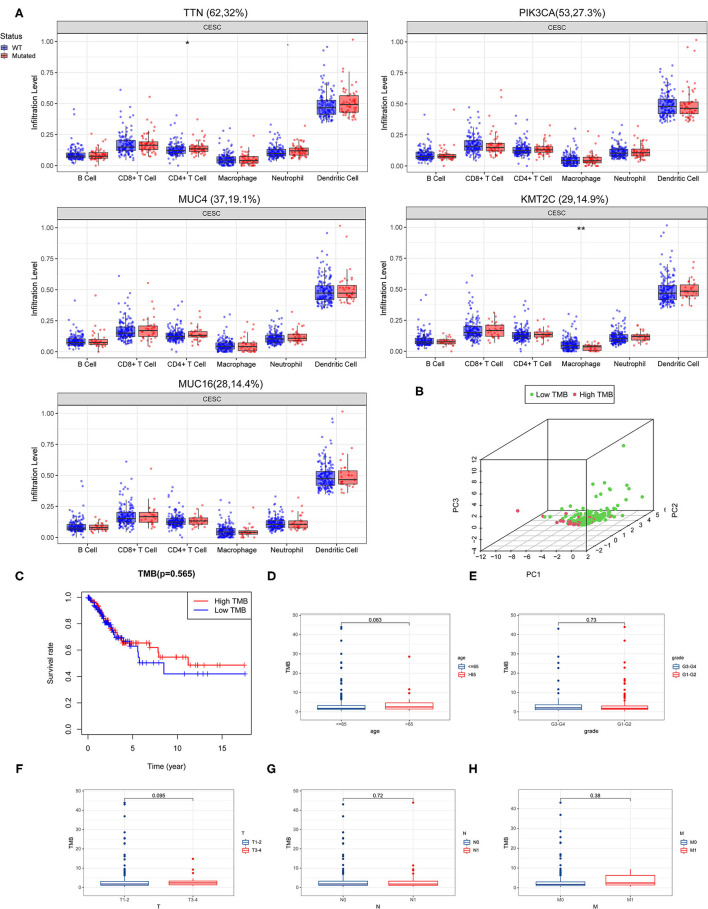
Correlation of tumor mutation burden (TMB) with survival outcomes and clinical parameters. **(A)** Association of the top five mutant genes with immune cell infiltration in the CESC microenvironment. **(B)** Principal component analysis (PCA) plot representing the distinction between the high and low TMB groups. **(C)** High TMB group was associated with higher overall survival. **(D–H)** TMB correlated with clinical parameters.

We evaluated the potential relationships of the top five mutant genes of CESC with immune cell infiltration in the CESC microenvironment. The results showed that there were significant differences in the level of immune infiltrates between wild-type (WT) and mutated genes (Figure 2A). Specifically, TTN mutations were associated with differences in CD4+ T cell levels, while for KMT2C, mutations were associated with differences in macrophages levels.

By calculating the number of mutated bases per million bases, the TMB was defined for 288 CESC samples. According to the median of TMB, patients with CESC were divided into the high TMB group and the low TMB group. The principal component analysis (PCA) revealed that genes in the high- and low-TMB groups were well separated (Figure 2B). However, Kaplan–Meier survival curve analysis revealed that although there was no significant difference between the high and low TMB groups, the trend of the 5-year survival rate of the high TMB group was higher than that of the TMB group (Figure 2C).

Next, we performed a correlation analysis between TMB and clinical parameters, and found that women over age 65 had higher TMB levels (Figure 2D). Moreover, grade T3-4 tumors had higher TMB levels than grades 1 and 2 tumors, grade T3-4 had higher TMB levels than grades T1 and T2, N0 had higher TMB levels than N1, and that M1 tumors had higher TMB levels than M0 tumors (Figures 2E–H).

### Comparison of Gene Expression Profiles in Different TMB Groups

**Figure 3:**
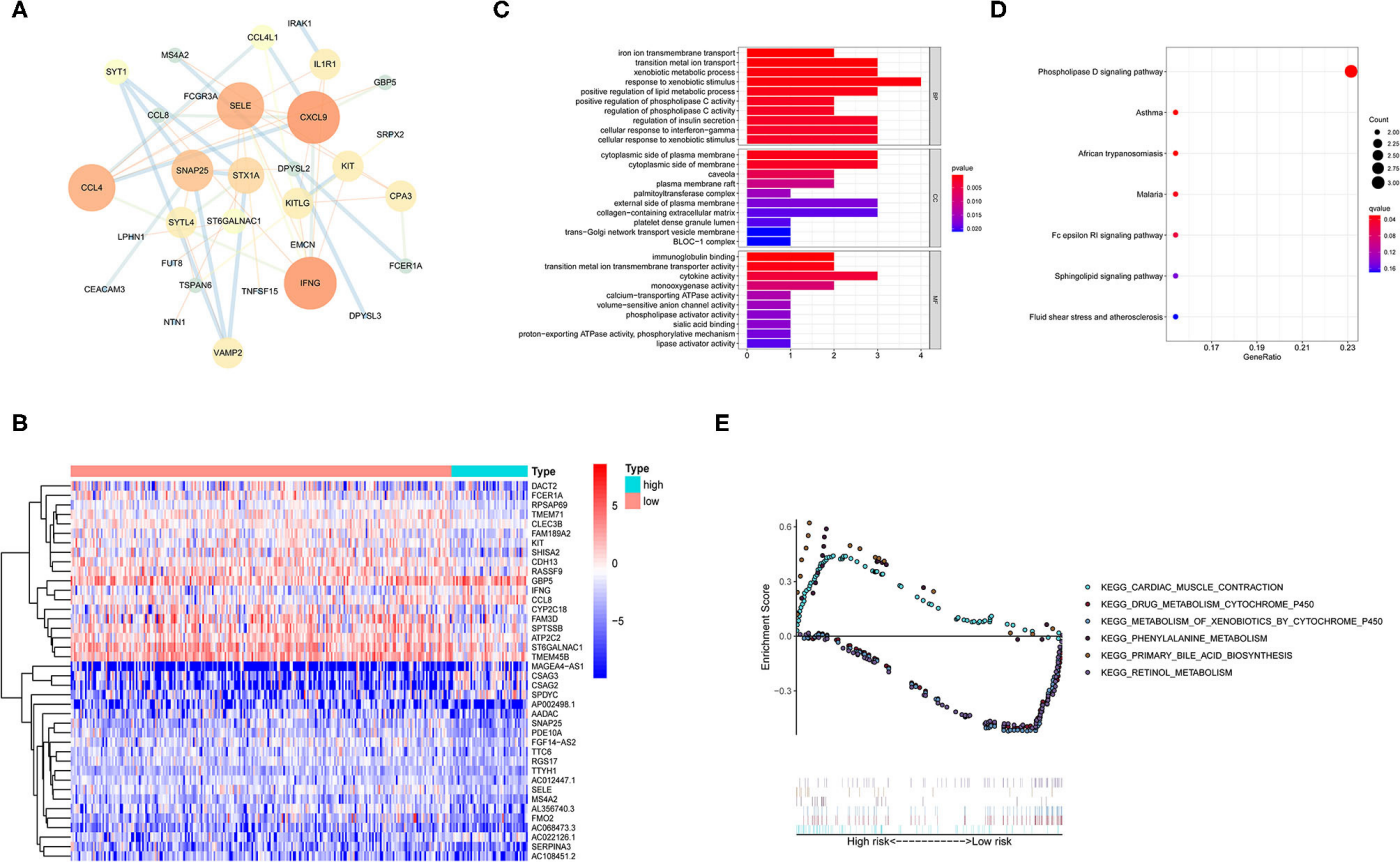
Functional enrichment and enrichment pathway analysis of differentially expressed genes (DEGs). **(A)** Protein-protein interaction (PPI) network analysis of the DEGs. **(B)** Heatmap representing the top 40 DEGs. **(C)** Gene Ontology (GO) enrichment analysis revealing the functional enrichment terms of DEGs. **(D)** Kyoto Encyclopedia of Genes and Genomes (KEGG) pathway enrichment analysis showing the enriched pathways of DEGs. **(E)** Gene set enrichment analysis (GSEA) analysis representing the TMB-related KEGG pathways. Above the horizontal axis indicated the pathways are in the high TMB group, and below the horizontal axis indicated that the pathways are in the low TMB group.

Differences in gene expression between the high TMB group and low TMB group were analyzed to identify differentially expressed genes (DEGs). Filter condition was |log fold change (FC)| > 1, and false discovery rate (FDR) was < 0.05. A total of 99 DEG were identified by screening. In addition, the protein-protein interaction (PPI) network of DEGs was further compiled using the STRING database and included 31 nodes and 48 edges (Figure 3A). After the genes were sequenced by “degree,” it was found that the degree value of IFNG (*n* = 8) and CXCL9 (*n* = 8) were the top two, suggesting that both genes participated in most biological functions. Meanwhile, the top 40 DEGs are revealed in a heatmap (Figure 3B).

### Functional Enrichment and Enrichment Pathway Analysis of DEGs

Gene ontology enrichment analysis was utilized to determine the function of DEGs (Figure 3C). In the biological process (BP) classification, the genes were significantly enriched in the “cellular response to interferon-gamma,” “positive regulation of lipid metabolic process,” and “xenobiotic metabolic process” terms. In the cellular component (CC), enrichment was mainly reflected in terms of “collagen-containing extracellular matrix,” “palmitoyltransferase complex,” “trans-Golgi network transport vesicle,” and “cytoplasmic side of membrane.” The enriched molecular function (MF) terms included “immunoglobulin binding,” “cytokine activity,” “calcium-transporting ATPase activity,” and “calcium-transporting ATPase activity.” These findings suggested that the DEGs were firmly related to immune response in the tumor microenvironment.

Kyoto Encyclopedia of Genes and Genomes pathway enrichment analysis was performed to define the pathways that were significantly enriched DEGs (Figure 3D). The KEGG results showed that the DEGs were involved in “Phospholipase D signaling pathway,” “Fc epsilon RI signaling pathway,” and “Sphingolipid signaling pathway.”

Furthermore, TMB-related Kyoto Encyclopedia of Genes and Genomes (KEGG) pathways were evaluated by gene set enrichment analysis (GSEA) (Figure 3E). The results showed that the high TMB group was mainly enriched in “phenylalanine metabolism,” “primary bile acid biosynthesis,” and “cardiac muscle contraction.” Similarly, “metabolism of xenobiotics by cytochrome p450,” “drug metabolism cytochrome p450,” and “retinol metabolism” gene sets were mainly active in the low TMB group.

Overall, the high TMB group was mainly associated with amino acid metabolism and lipid metabolism, while the low TMB group was mostly associated with xenobiotic and drug metabolism.

### Differential Infiltration of Tumor-Infiltrating Immune Cells in Different TMB Groups

**Figure 4:**
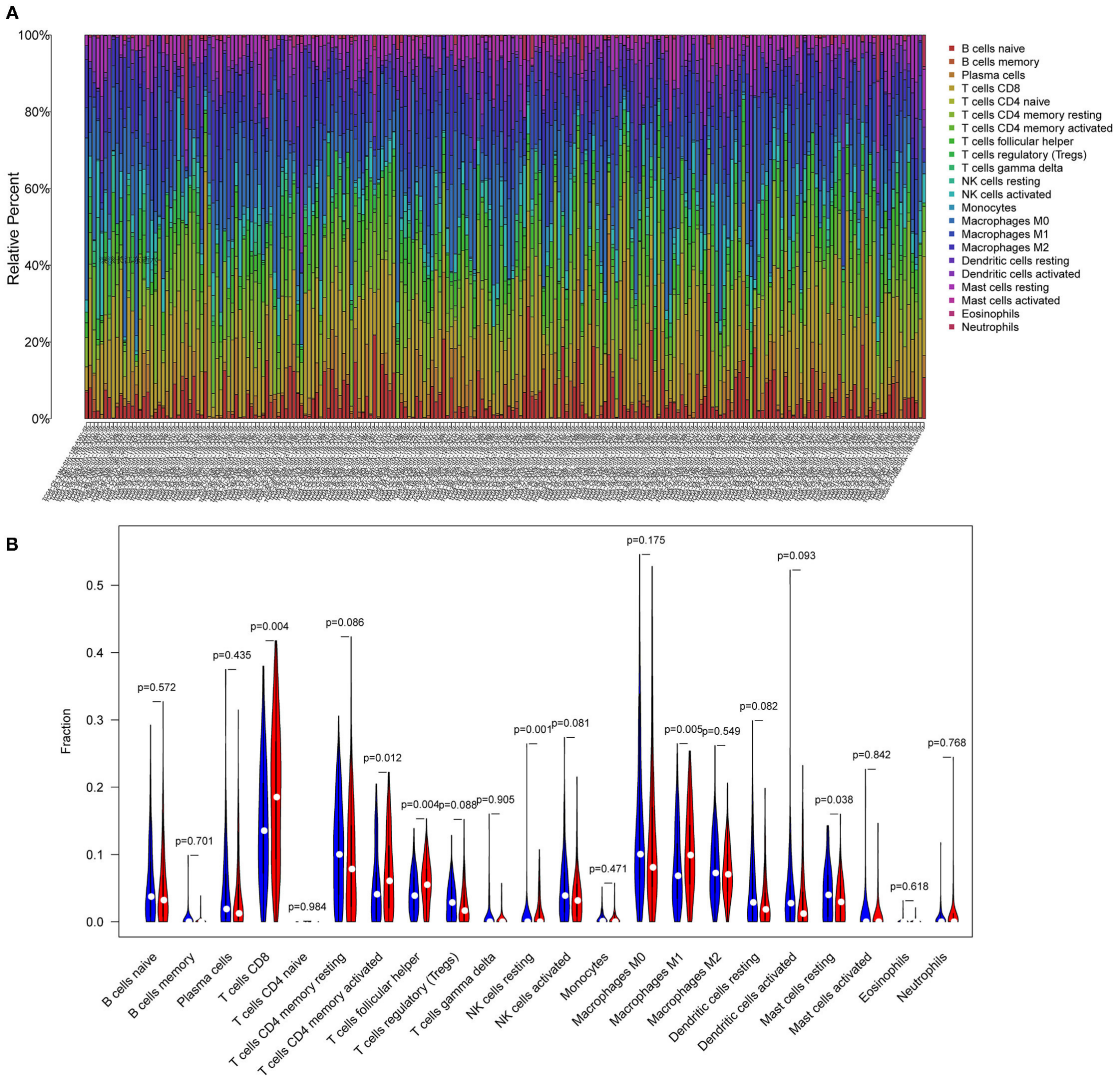
Differential infiltration of tumor-infiltrating immune cells in different TMB groups. **(A)** Stacked bar plot representing the relative proportion of 22 immune cell types in each sample. **(B)** Violin plot showing the differential infiltration of 22 immune cells between the high TMB and low TMB groups. In red, the high TMB group and in blue, the low TMB group.

The CIBERSORT algorithm was applied to evaluate the distribution of different tumor-infiltrating leukocytes (TILs) between the high and low TMB groups. A *P* < 0.05 indicated that the CIBERSORT algorithm had high accuracy in calculating the relative percent of TIL cells. As shown in Figure 4A, the distribution of different immune cells in each sample varies significantly. Furthermore, the high TMB group was significantly correlated with high fractions of CD8+ T cells, activated memory CD4+ T cells, follicular helper T cells, and M1 macrophages. For the low TMB group, there was a higher fraction of resting NK cells and resting mast cells (Figure 4B).

### Identification of PIRDEGs

**Figure 5:**
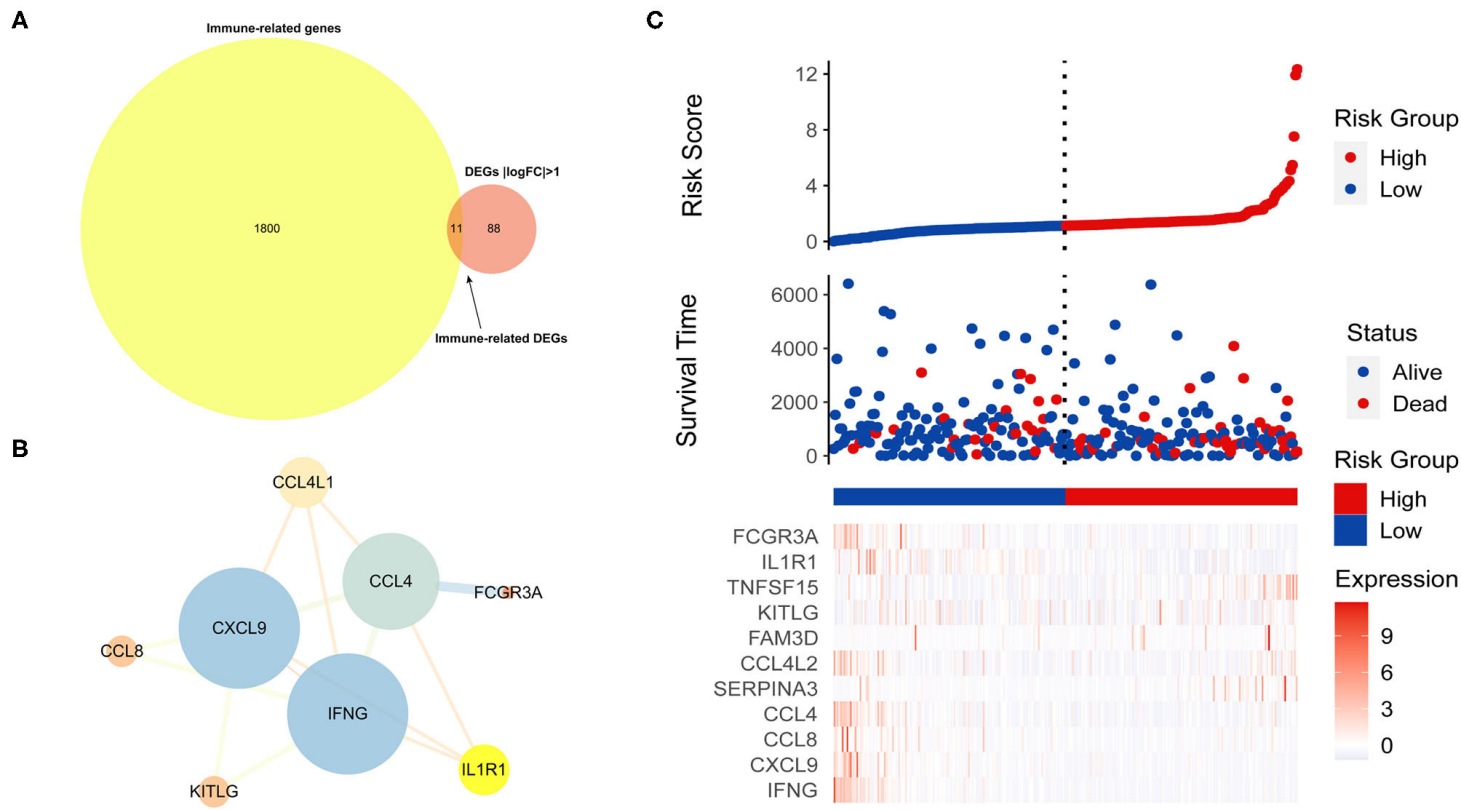
Identification of immune-related differentially expressed genes related to prognosis (PIRDEGs). **(A)** Venn plot displaying the 11 IRDEGs. **(B)** The PPI network of IRDEGs. **(C)** The graph presents the risk score distribution of patients, survival status scatter plots of patients, and expression levels of risk genes.

Immune-related genes were downloaded from the IMMPORT database, and 11 immune-related DEGs (IRDEGs) were obtained from the intersection of 1,811 immune-related genes (IRGs) and 99 DEGs (Figure 5A). Next, the PPI network of IRDEGs was merged further using the STRING database. In the plot obtained, the average node degree was 3.5, with eight nodes and 14 edges (Figure 5B).

Next, we used patients from the TCGA-CESC dataset to produce a training cohort and evaluate the relationship between the TMB and risk score. The patients were divided into the high-risk (*n* = 48) and low-risk (*n* = 240) groups by the median risk score and then were ranked according to the risk score. The results indicated that the TMB was positively correlated with risk score. The dot chart revealed the survival state of the patients, and the heatmap described the expression levels of the IRDEGs in the different groups (Figure 5C).

### Construction and Verification of the Prognostic Model

The multivariate Cox regression analysis identified five hub genes that were used to build a prognostic model. The five hub genes included in this model were interferon gamma (IFNG), serpin family A member 3 (SERPINA3), C-C motif chemokine ligand 4 like 2 (CCL4L2), TNF superfamily member 15 (TNFSF15), and interleukin 1 receptor type 1 (IL1R1). The risk score was calculated as follows:

Risk score = (−0.530 × expression of IFNG) + (0.166 × expression of SERPINA3) + (0.095 × expression of CCL4L2) + (0.343 × expression of TNFSF15) + (−0.056 × expression of IL1R1).

**Figure 6:**
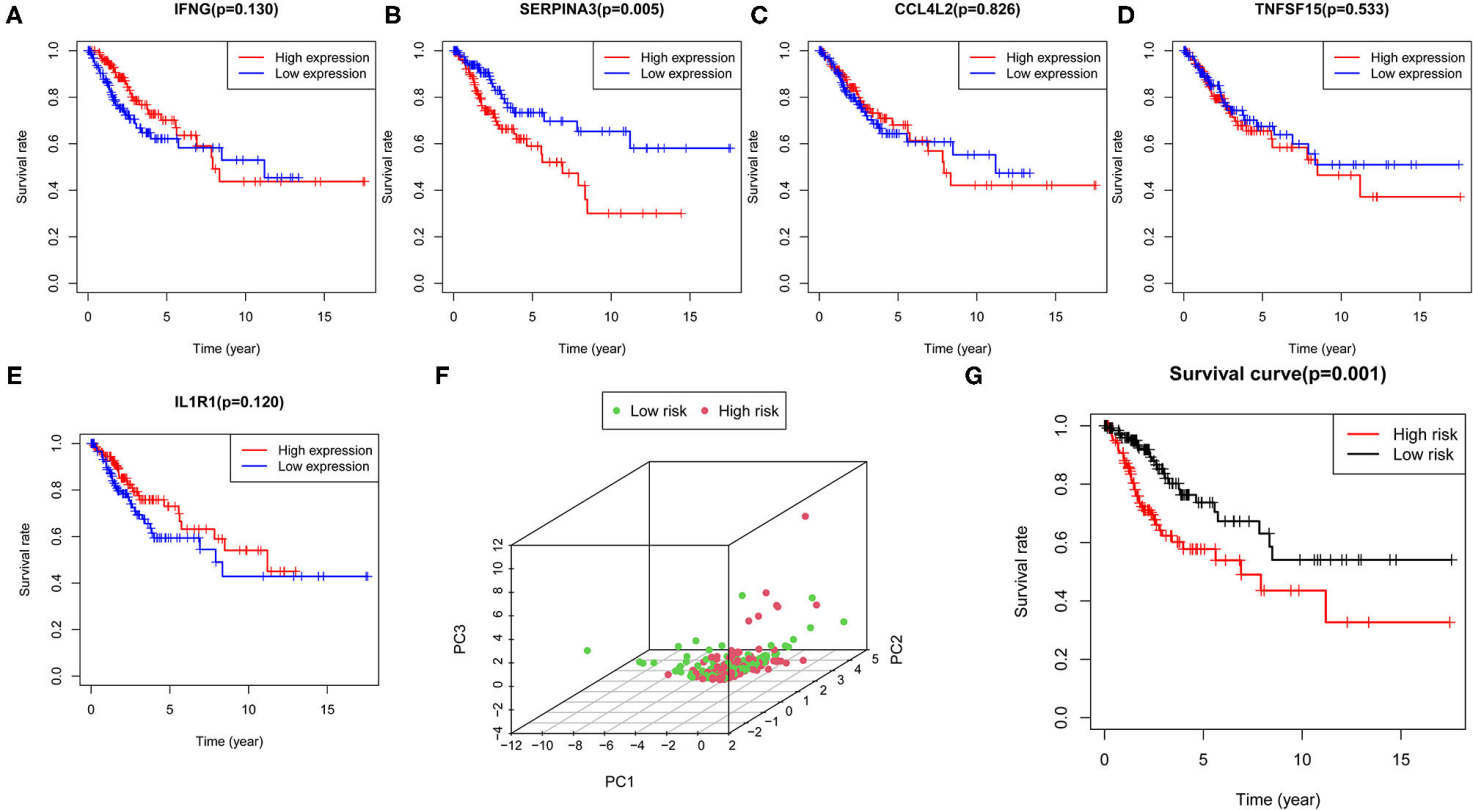
Construction of the prognostic model. **(A–E)** Correlation of the expression levels of five hub genes with survival outcomes. **(F)** PCA plot represents the distinction between the high- and low-risk groups. **(G)** Survival curve indicating significant differences in survival outcomes between the high- and low-risk groups.

Based on the median value of the confidential intervals (CIs), the patients were stratified into the high expression group and low expression group, and the correlation between the expression levels of the five hub genes and OS was analyzed (Figures 6A–E). The high expression of most of the hub genes indicated a poor prognosis.

Next, we determined the risk score value for each patient, and then the patients were stratified into the high- and low-risk groups *via* the median value of the risk score. The PCA plot was used to distinguish between the high- and low-risk groups (Figure 6F). The survival curves showed the survival rate between the high- and low-risk groups had significant differences, and that the patients in the low-risk group had a longer survival rate than those in the high-risk group (Figure 6G). Furthermore, we performed Cox regression analysis to determine whether the risk score generated by the prognostic model was independent of other clinical parameters. The results indicated that T, M, N, and the risk score were significantly correlated with OS (*P* < 0.05) (Supplementary Figure 2), which suggested that the risk model could serve as a prognostic factor independent of other clinical parameters.

**Figure 7:**
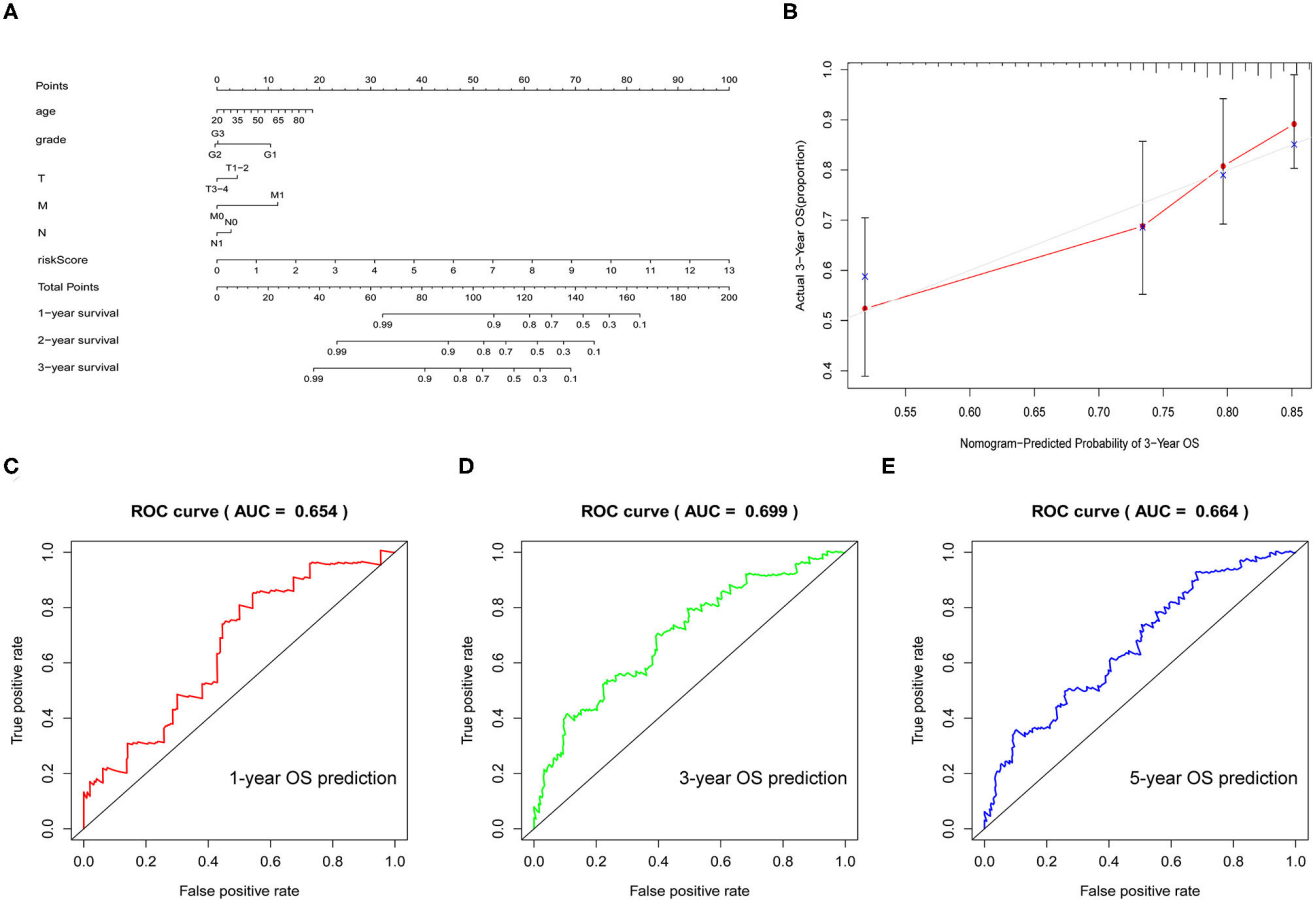
Verification of the prognostic model. **(A)** Nomogram was used to predict the 1-, 3-, and 5-year survival rates of patients with CESC. **(B)** Nomogram-predicting 3-year overall survival (OS). **(C–E)** Receiver operating characteristic (ROC) curve used to analyze the accuracy of the prediction model.

To better predict the prognosis of patients with CESC, we established a nomogram model that could accurately predict the OS at 1, 3, and 5 years based on the variables associated with OS (age, grade, TMN, and risk score) (Figure 7A). The calibration curve was constructed as a nomogram to predict the 3-year OS to examine the accuracy of the nomogram. The results suggested that the nomogram could accurately predict the OS of patients with CESC (Figure 7B). Moreover, receiver operating characteristic (ROC) curve analysis was explored to assess the accuracy of the prediction model. The area under the curve (AUC) for the 1-, 3-, and 5-year OS was 0.654, 0.699, and 0.664, respectively (Figures 7C–E).

In short, this model could accurately predict the survival time of patients and had a very practical clinical value.

### Correlation of the Five Hub Genes With Immune Cell Infiltration

**Figure 8:**
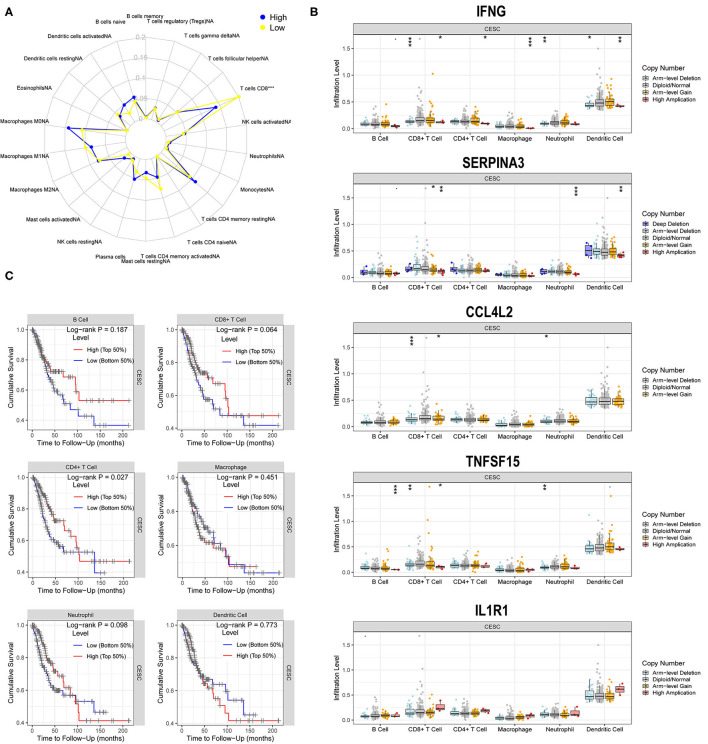
Correlations of five hub genes with immune cell infiltration. **(A)** Radar plot showing differences in immune cell infiltration between the high- and low-risk groups. *, *P* < 0.05; **, *P* < 0.01; ***, *P* < 0.001. **(B)** Box plots presenting the distributions of six immune cells in different copy number status of five hub genes in CG. **(C)** Kaplan–Meier analysis revealing low infiltration levels of CD4+T cell associated with poor prognosis in CESC (*P* < 0.05).

Based on the radar plot, the infiltration of CD8+T cells showed significant differences between the high- and low-risk groups (Figure 8A). Thus, we evaluated the potential relationships between different copy number status of the five hub genes (IFNG, SERPINA3, CCL4L2, TNFSF15, and IL1R1) and immune cell infiltration levels in the CESC microenvironment. As shown in Figure 8B, different mutations harbored by the five hub genes are mainly associated with the infiltration level of CD8+ T cells and dendritic cells (DCs).

A multivariate Cox regression model was used to further investigate the potential prognosis of immune cells in the CESC samples (302 patients with 73 deaths). A high level of CD8+Tcell infiltration was associated with CESC prognosis and was a risk factor for CESC (Table 1). In addition, Kaplan-Meier analysis revealed that low infiltration levels of CD8+T cells were associated with poor prognosis in CESC (Figure 8C).

**Table 1:** Multivariate Cox regression analysis of immune infiltration cells in cervical squamous cell carcinoma (CESC).

**Immune cells**	**Coeff**	**HR**	**Lower 95% CI**	**Upper 95% CI**	***P*-value**	**Sig**.
B cell	−1.872	0.154	0.000	1324.554	0.686	-
CD8 T cell	−4.799	0.008	0.000	0.961	0.048	*
CD4 T cell	−5.688	0.003	0.000	7.542	0.148	-
Macrophage	1.979	7.236	0.006	8707.998	0.584	-
Neutrophil	−2.622	0.073	0.000	631.194	0.571	-
Dendritic	3.699	40.399	0.372	4388.085	0.122	-

*R square = 0.036 (max possible = 9.04e-01); likelihood ratio test p = 8.55e-02; Wald test p = 2e-01; score (log-rank) test p = 2.09e-01*.

**Figure 9:**
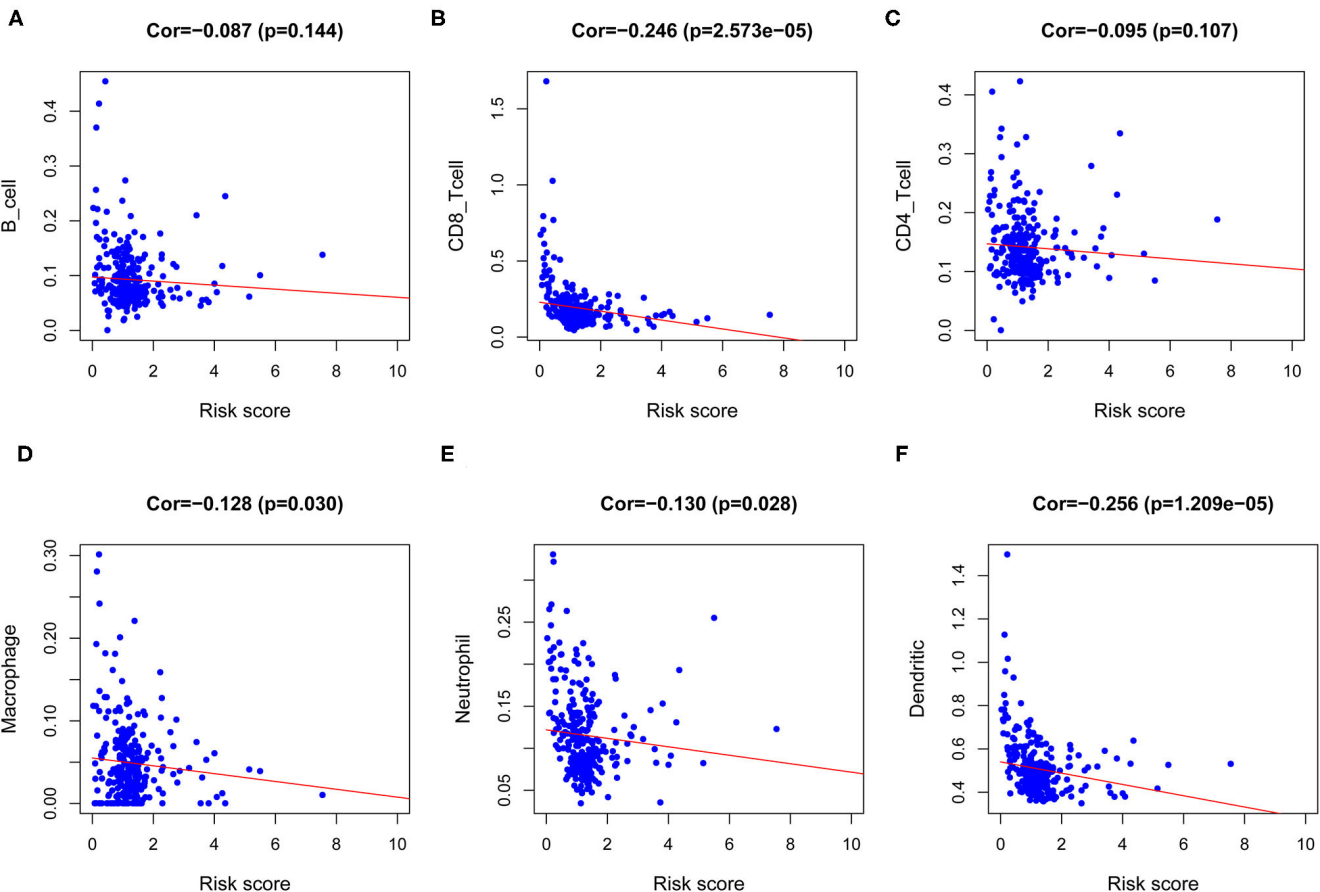
Analysis of the correlation between the risk score and immune cells infiltration. **(A)** Correlation between risk score and B cell. **(B)** Correlation between risk score and CD8 T cell. **(C)** Correlation between risk score and CD4 T cell. **(D)** Correlation between risk score and Macrophage. **(E)** Correlation between risk score and Neutrophil. **(F)** Correlation between risk score and Dendritic cell.

Ultimately, to identify whether the prognostic model could reflect the tumor immune microenvironment status of patients with CESC, we analyzed the association between risk score and immune infiltrates. The results indicated that risk score had a negative correlation with the abundance of immune cells in the CESC microenvironment (Figures 9A–F), which indicated that with higher risk scores, the levels of immune cells in the microenvironment of CESC decreased.

**Figure 10:**
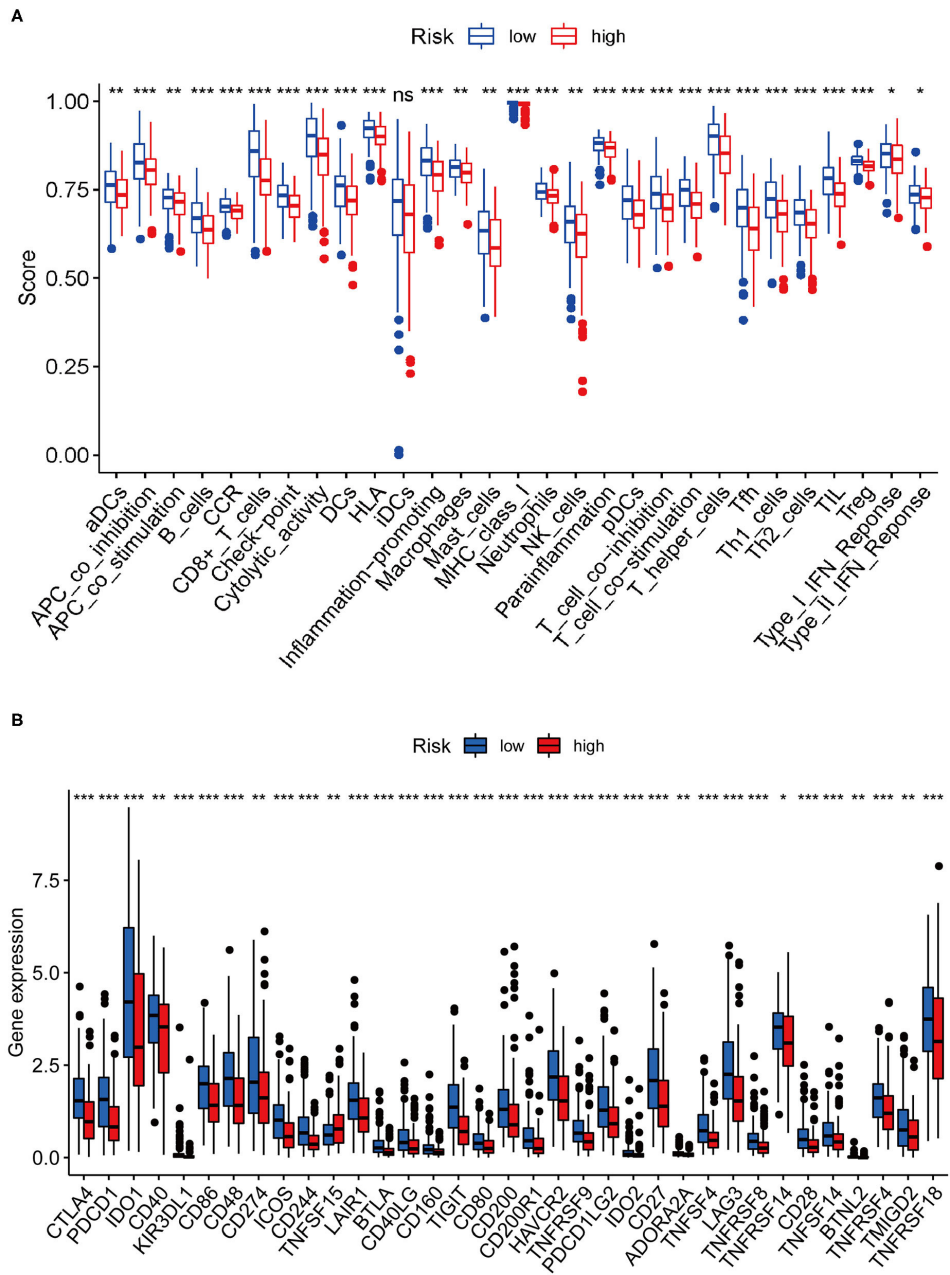
Correlation between risk score and immune-related functions and immune checkpoint molecule expression. **(A)** Scores of immune cells and immune-related functions between the high- and low-risk groups. **(B)** Differences in the expression of immune checkpoint molecules between the high- and low-risk groups.

We performed single-sample GSEA (ssGSEA) analysis to determine the correlation between immune cell subsets and related functions. The results revealed that the scores associated with the T-cell inflammation signature (TIS) and tumor immune dysfunction and exclusion (TIDE) profiles of immune checkpoints in the low-risk group were higher than those in the high-risk group, such as APC co-inhibition, cytokine–cytokine receptor (CCR), CD8+ T cells, Check-point, Cytolytic activity, HLA, Inflammation-promoting, MHC class I, Parainflammation, T cell co-inhibition, T cell co-stimulation, T helper cells, Tfh, Th1 cells, Th2 cells, TIL, Treg, Type I IFN response, and Type II IFN response (Figure 10A). The expression of immune checkpoint molecules was closely associated with mechanisms involved in immune escape. Given the importance of ICB-based immunotherapy, we explored differences in the expression of immune checkpoint molecules between the high- and low-risk groups. The results showed that the expression level of common immune checkpoint molecules, such as IDO1, LAG3, CTLA4, TIGIT, CD86, PDCD1, CD48, and CD44 (Figure 10B) in the low-risk group was higher than that in the high-risk group.

## Discussion

Cancer is a genetic disease. The accumulation of somatic copy number alterations (SCNA) leads to tumors. Genetic alterations include driver mutations that have a direct effect on tumor growth and passenger mutations that indirectly affect cancer cell growth (15–17). In addition, there are significant differences in the frequency of gene alterations (including missense mutations, synonymous mutations, insertions or deletions, and copy number gains and losses) across different tumor types (15, 18–20). The TMB may serve as an indicator to predict the clinical outcome of ICB and has become a valuable biomarker in many solid cancers for determination of patients who may benefit from immunotherapy (21–24). Several studies have confirmed that PD-L1 is upregulated in CESC and is correlated with a better survival rate (12, 25). However, the prognostic role of TMB in CESC and its relevance to immunotherapy have not been explored. Herein, we explored the prognostic function of TMB in CESC and its underlying relevance in immune infiltration.

Considering the landscape of gene mutations across CESC samples, we observed that missense mutations were the most frequent, and that SNP was the most common type of mutation. TTN, PIK3CA, KMT2C, MUC16, and MUC4 were identified as the most frequently mutated genes of CESC. Moreover, there is a strong co-occurrence relationship among mutant genes. Next, the study found that there were significant differences in the level of immune infiltrates between the wild-type and mutated genes among the top five mutated genes. The Kaplan-Meier survival curve analysis indicated that patients with high TMB had a prolonged 5-year survival time compared with those with low TMB. Based on the results of Phase II TMB data, patients in the high TMB group with at least 243 missense mutations in tumors and treated with nivolumab therapy had significantly improved progression-free survival (PFS) vs. standard of care (SOC) chemotherapy. Strikingly, PD-L1 expression among all patients subjected to the TMB analysis also exceeded 1% (21, 26). Similarly, Lauss et al. found that tumor mutation and new antigen load can predict the improvement of PFS and OS in patients with melanoma treated with adoptive T cell metastasis (27). Negrao et al. observed that low TMB and the negative expression of PD-L1 were predictors of poor prognosis in non-small cell lung cancer (28).

Next, we identified 99 DEGs between the high and low TMB groups. The results of the GO and KEGG analyses, along with those of the GSEA, suggested that the DEGs were associated with the tumor immune microenvironment and metabolism. By evaluating the distribution of 22 infiltrating immune cells between the high and low TMB groups, the distribution of different immune cell subtypes in each sample varied significantly. Furthermore, the high TMB group was significantly correlated with high fractions of CD8+ T cells, activated memory CD4+ T cells, follicular helper T cells, and M1 macrophages. For the low TMB group, there was a higher fraction of resting NK cells and resting mast cells. The tumor microenvironment (TME) is closely associated with the response to ICB treatment, and the abundance of tumor-infiltrating CD4+T cells and CD8T+ cells are associated with the immune response (29, 30). It has been reported that the expression of PD-L1 and the density of CD8 + T cells cannot only be used as independent prognostic factors, and that the correlated expression between PD-L1 and CD8 + T cells is also related to the PFS or OS in CESC (25). In addition, tumor-infiltrating NK cells are associated with a favorable prognosis in patients with cancer (31). A total of 11 IRDEGs were obtained from the intersection between 1,811 IRGs and 99 DEGs. Next, univariate Cox and multivariate Cox regression analyses were performed to identify five hub genes (IFNG, SERPINA3, CCL4L2, TNFSF15, and IL1R1) and build a prognostic model. The high expression of most genes in this prognostic risk model indicated a worse prognosis. Our findings revealed that the low-risk patients with CESC tended to be immunologically “hot” and were more likely to benefit from ICB therapy, and that the high-risk patients tended to be immunologically “cold” and would less likely benefit from ICB therapy. Overall, our prognostic risk model could predict the efficacy of ICB therapy for patients with CESC.

Recent research based on a Phase III melanoma immunotherapy trial showed that high TMB and high IFNG-related gene expression signature score were associated with pathological response and low-risk of recurrence (32). Various clinical trials have demonstrated that the high expression of SERPINA3 is an independent prognostic factor of the OS rate of patients with glioblastoma, and that it is associated with poor prognosis and tumor recurrence (33, 34). Ko et al. reached a similar conclusion in liver cancer (35). The CCL4L2 gene exists as a population-specific multiple copy number, which can reflect CNV between individuals (36). CCL4L2 is generally regarded as a pro-inflammatory factor and stimulates the transport of T helper 1 cells, regulatory T cells, monocytes, and dendritic cells (37). The TNFSF15 gene is involved in the development of a variety of cancers. For colon cancer, the high expression of TNFSF15 gene isoforms was related to tumor progression and encoded two protein molecules (TL1A and VEGI-192), which can be considered as independent prognostic factors (38). In addition, some TNFSF15 SNPs have been considered gastric cancer risk factors (39). Takahashi et al. reported that the overexpression of IL-1β in KRAS G12D mutant mice could activate IL-1R1-mediated epithelial cell proliferation and increase the level of immunosuppressive PD-L1+ B cells through the autocrine pathway, thereby inducing the progression of pancreatic ductal adenocarcinoma (40).

In our prognostic model, the survival curve showed that the low-risk group had better OS than the high-risk group. This result indicated that the model may be used to differentiate patients who will achieve a different response to immunotherapy, and, that the model could make individualized therapy possible. The correlation between the TMB and clinical parameters revealed the potential of TMB as a predictive biomarker in various tumors (4, 6). To better predict the prognosis of patients with CESC, we built a nomogram model to predict the OS at 1, 3, and 5 years based on the clinical variables and risk score, and the prediction results were verified by the ROC curve. Taken together, this model could accurately predict the survival time of patients and had a very practical clinical value.

Furthermore, we observed that a fraction of TILs in the TME of CESC presented significant differences between the high- and low-risk groups. Subsequently, we found that mutations of various forms carried by the five hub genes mainly affected the infiltration level of CD8+ T cells and DCs. Tsujitani et al. found that postoperative adjuvant immunotherapy could prolong the survival of patients with gastric cancer and fewer DCs (41). It is well known that the presence of tumor-infiltrating lymphocytes is generally considered to be a good prognostic factor for many cancers. Clinical studies have shown that patients with PD-L1 expressing tumors lacking CD8+ intra-tumoral cells have a clear trend of shorter PFS (25). In our study, the high level of CD8+T cell infiltration was related to the prognosis of CESC and was a risk factor for CESC. This was consistent with previous studies. We further assessed the relevance of risk score and immune infiltrates, and showed that the content of immune cells increased as the risk score decreased in CESC.

In summary, this study indicated that patients with CESC in the high TMB group have a favorable prognosis. Our results provide evidence to improve our understanding of the relevance of the TMB and TILs in CESC, which may be valuable for exploring the role of the TMB in CESC. Furthermore, this study identified an accurate risk model for predicting the prognosis of CESC using five PIRDEGs based on the TCGA cohort. This model can be used to stratify patients with different TMB, and will help to predict the sensitivity of patients to immunotherapy. Moreover, our prognostic model may reflect immune infiltrates present in the TME and will provide underlying biomarkers for personalized immunotherapy. However, additional experimental studies and larger sample clinical trials are needed to validate the findings of this study.

## Methods

### Data Collection

The transcriptome profiles, related clinical data, and somatic mutation data of patients with CESC were downloaded from the TCGA data portal (https://portal.gdc.cancer.gov/). Perl scripts were used to extract and collate transcriptome data and clinical information. Mutation data were analyzed by the Varscan software. The R package “maftools” was used to visualize mutation data. Moreover, the list of IRGs was obtained from the IMMPORT database (https://www.immport.org/), which is extensively utilized in immune-related studies (42, 43).

### Grouping and Clinical Analysis of TMB

The TMB was calculated as the number of mutated bases per million bases. In this study, the Perl script was used to calculate mutation frequency and the number of variants/exon length of each sample (38 million) (44). Based on the median value of TMB, the CESC samples were divided into the high TMB group and the low TMB group. The survival difference between the high and low TMB groups was compared by Kaplan-Meier analysis. The relationship between TMB levels and clinical characteristics was also evaluated. The Wilcox test was performed to compare the two groups of clinical variables, and the Kruskal test was performed to compare three or more groups of variables.

### Function and Pathway Analysis of DEGs

Differentially expressed genes between the high and low TMB groups were selected by the condition “|log FC| > 1 and FDR < 0.05,” and the DEGs were plotted with the R package *ggplot2* and *pheatmap*. The PPI network of DEGs was retrieved and constructed *via* the STRING database (https://string-db.org/) and using the Cytoscape software (https://cytoscape.org/). The R package *org.Hs.eg.db* was used to convert gene names into Entrez IDs for functional and pathway analysis. The functional analysis of GO terms and enrichment analysis of KEGG pathways were carried out using the R package components *colorspace, stringi, ggplot2, DOSE*, and *clusterProfiler*, as well as *enrichplot*, and setting *p* < 0.05 and *q* < 0.05 as the filtering conditions. Furthermore, GSEA was performed to analyze the KEGG pathways. The c2.cp.kegg.v6.2.symbols.gmt gene set was selected as the reference gene set. The significantly enriched KEGG pathways were obtained by filtering with FDR <25%.

### Analysis of the Relationship Between TMB and Immune Cells Infiltration

The CIBERSORT algorithm (R Script v1.03) was used to calculate the fraction of immune infiltrates in each sample. *P* < 0.05 indicated that the accuracy of using the CIBERSORT software to predict immune cell infiltration was high (45). The IRDEGs were extracted by the intersection of IRGs and DEGs with log |FC| > 1. The PPI network of IRDEGs was retrieved and constructed using the STRING database. The univariate Cox regression analysis was performed to identify IRDEGs that were closely related to prognosis. A *P* < 0.05 was considered to be associated with prognosis.

### Construction and Verification of Prognostic Model of IRDEGs

The multivariate Cox analysis was performed to optimize the model forward and backward to obtain the hub genes. The risk value of each patient was obtained according to the formula risk score. The risk score = (Coefficient_mRNA1_ × expression of mRNA1) + (Coefficient_mRNA2_ × expression of mRNA2) +…+ (Coefficient_mRNAn_ × expression of mRNAn). Next, the patients were stratified into high- and low-risk groups *via* the median value of risk. The R package scatterplot3d was applied to see if the high- and low-risk groups were well differentiated. The nomogram and ROC curve were explored to predict the prognosis of patients with CESC. An AUC >0.6 was considered reliable for predictions (46).

### Correlations of Five Hub Genes With Immune Cell Infiltration

The CIBERSORT results were filtered with *P* < 0.05 to extract the number of immune cells in the high- and low-risk groups, and the difference in the number of immune cells between the high- and low-risk groups was analyzed. Furthermore, we analyzed the correlation between hub genes with immune cell infiltration in the CESC based on the TIMER database (https://cistrome.shinyapps.io/timer/). The SCNA module analysis was performed for the comparison of tumor infiltration levels with various SCNA for the hub genes. The infiltration level of each SCNA category is compared with the normal values by the two-sided Wilcoxon rank-sum test. The Survival module was used to analyze the clinical relevance of six tumor immune subsets in a multivariable Cox proportional hazard model. Surv (STAD) ~variables (different tumor infiltrating leukocytes) were used as the formula applied for the Cox's regression model. The Mutation module was applied to compare the levels of immune infiltrates with or without the presence of a given mutation. In CESC, we chose the top five genes to compare the distribution of immune infiltration levels under different gene mutation statuses, with statistical significance estimated by the two-sided Wilcoxon rank-sum test.

### Statistical Analysis

The R software (version 3.6.1, https://cran.r-project.org/bin/windows/base/) was applied to perform all the statistical analyses. The Wilcoxon test was suitable for the comparison of data between two groups, and the Kruskal-Wallis test was performed to compare three or more groups. The survival data were analyzed by Kaplan-Meier curves and log-rank tests. The univariate Cox regression analysis was performed to determine the PIRDEGs. The multivariate Cox regression analysis was performed to determine the hub genes and to investigate the potential prognosis of immune cells in the CESC samples. For all the comparisons in this study, a two-tailed *P* < 0.05 was considered statistically significant.

## Data Availability Statement

Publicly available datasets were analyzed in this study. This data can be found at: https://portal.gdc.cancer.gov/.

## Author Contributions

FW and PS conceived, designed, and wrote the manuscript. SR and WH analyzed the data and generated the figures and tables. XC and YW performed literature search and collected data for the manuscript. SG revised the manuscript. JL and SL revised the images. PS provided scientific research fund support. All authors have read and approved the final manuscript.

## Funding

This study was supported by the Pilot Gastric Cancer Project of Clinical Cooperation of Traditional Chinese and Western Medicine for Major and Difficult Diseases, The Open Projects of the Discipline of Chinese Medicine of Nanjing University of Chinese Medicine Supported by the Subject of Academic Priority Discipline of Jiangsu Higher Education Institutions (No. ZYX03KF020), and National Administration of Traditional Chinese Medicine: 2019 Project of Building Evidence-Based Practice Capacity for TCM (No. 2019XZZX-ZL003).

## Conflict of Interest

The authors declare that the research was conducted in the absence of any commercial or financial relationships that could be construed as a potential conflict of interest.

## Publisher's Note

All claims expressed in this article are solely those of the authors and do not necessarily represent those of their affiliated organizations, or those of the publisher, the editors and the reviewers. Any product that may be evaluated in this article, or claim that may be made by its manufacturer, is not guaranteed or endorsed by the publisher.

## Supplementary Material

Supplementary Figure 1 Mutation annotation format transition and transversion visualization in CESC.

Supplementary Figure 2 Independent prognostic value of the prognostic risk model. **(A)** Forrest plots of the univariate Cox regression analysis. **(B)** Forrest plot of the multivariate Cox regression analysis.

Supplementary Table 1 Clinical information of patients with cervical squamous cell carcinoma (CESC).

## Abbreviations

CESC: cervical squamous cell carcinoma
SNP: single nucleotide polymorphism
SNV: single nucleotide variants
TMB: tumor mutation burden
TCGA: The Cancer Genome Atlas
CI: confidential interval
DEGs: differentially expressed genes
FC: fold change
FDR: false discovery rate
BP: biological process
CC: cellular component
MF: molecular function
GO: gene ontology
KEGG: Kyoto Encyclopedia of Genes and Genomes
GSEA: Gene Set Enrichment Analysis
CNV: copy-number variation
IRGs: immune-related genes
IRDEGs: immune-related differentially expressed genes
PIRDEGs: immune-related differentially expressed genes related to prognosis
TMB: tumor mutational burden
HR: hazard ratios
OS: overall survival
WT: wild type
PPI: protein-protein interaction
ICB: immune checkpoint blockade
MMR: mismatch repair
DCs: dendritic cells
PD-L1: programmed death-ligand 1
PFS: progression-free survival
AUC: area under the curve
TME: tumor microenvironment
ROC: receiver operating characteristic
TILs: tumor-infiltrating leukocytes
PCA: principal component analysis.
