# Supplementary figures and images for: Prognostic Value of Tumor Mutational Burden Related to Immune Infiltration in Cervical Squamous Cell Carcinoma
Source: Front Med (Lausanne). 2021 Nov 11;8:755657. doi: 10.3389/fmed.2021.755657 (PMC8631969; doi:10.3389/fmed.2021.755657)

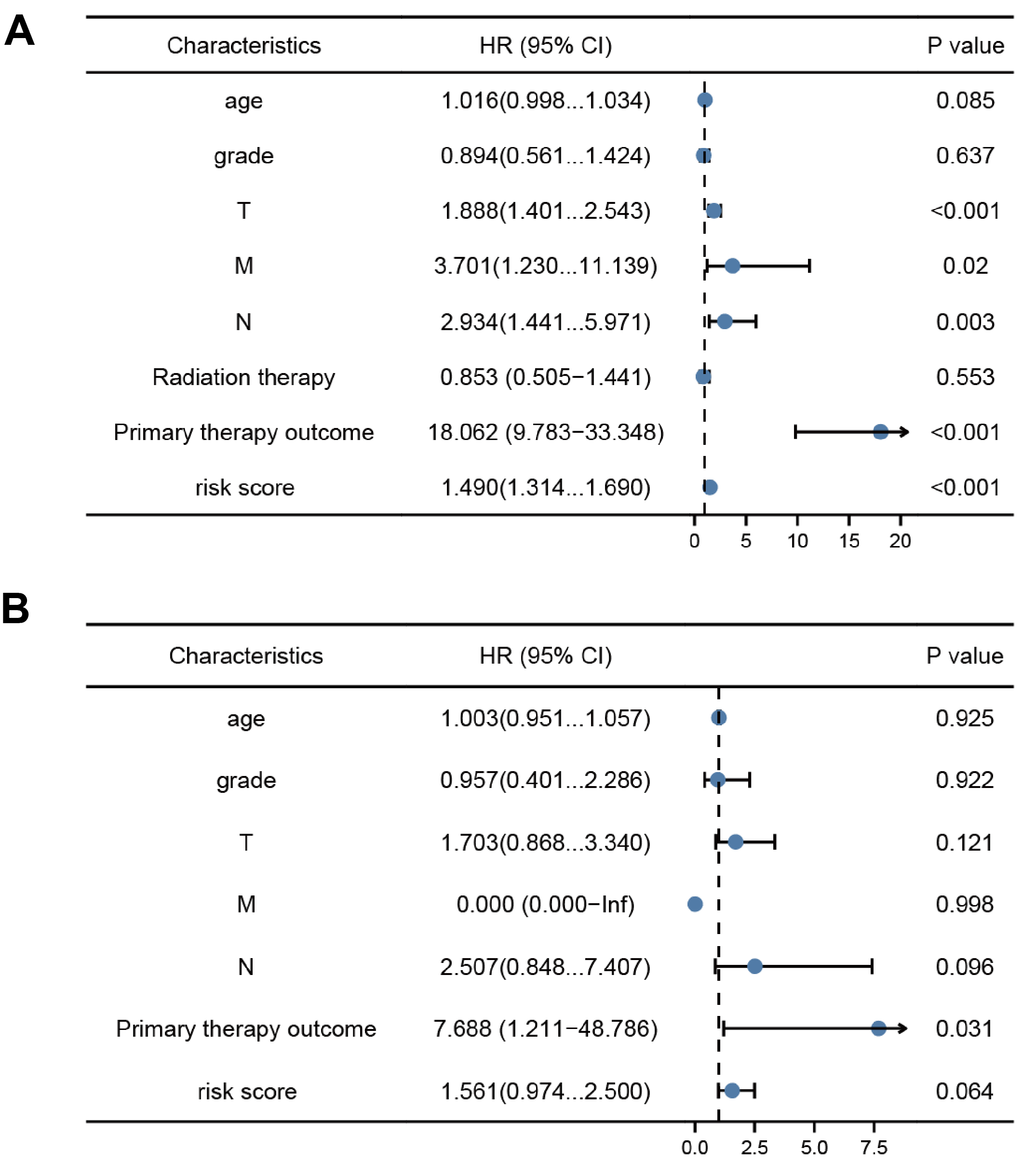

Supplement: Supplementary Figure 1 — Mutation annotation format transition and transversion visualization in CESC. [file Image_1.TIF]

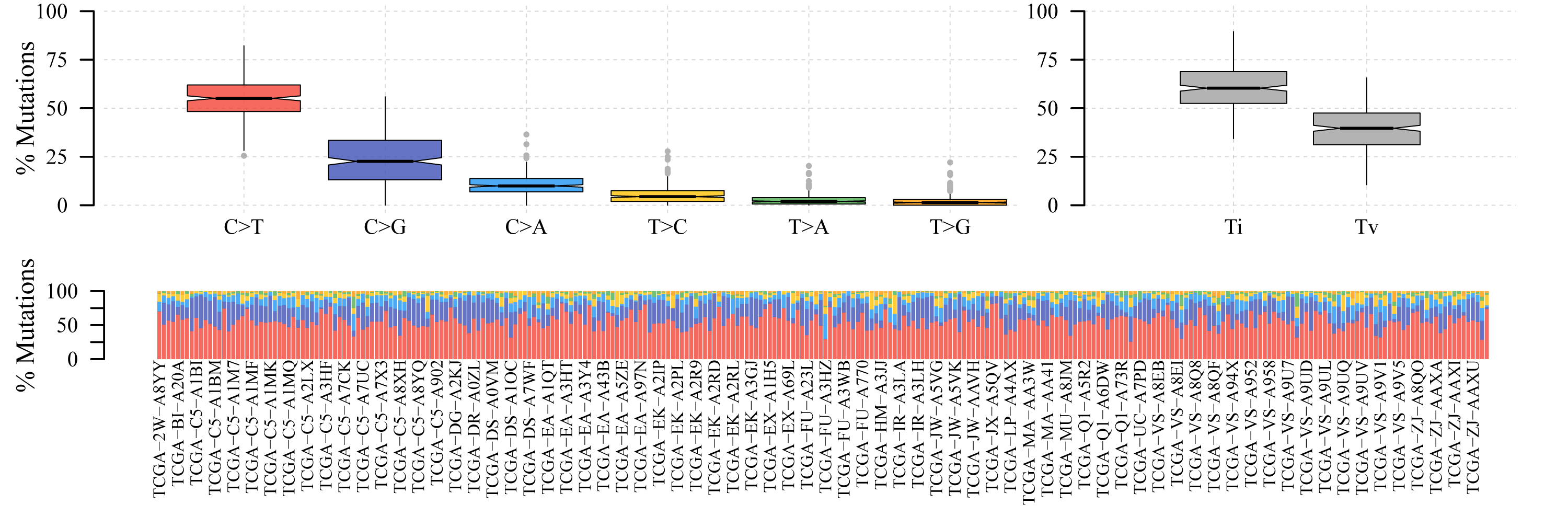

Supplement: Supplementary Figure 2 — Independent prognostic value of the prognostic risk model. (A) Forrest plots of the univariate Cox regression analysis. (B) Forrest plot of the multivariate Cox regression analysis. [file Image_2.TIF]
