# Supplementary material for: Prognostic Value of Tumor Mutational Burden Related to Immune Infiltration in Cervical Squamous Cell Carcinoma
Source: Front Med (Lausanne). 2021 Nov 11;8:755657. doi: 10.3389/fmed.2021.755657 (PMC8631969; doi:10.3389/fmed.2021.755657)
Supplement: Supplementary Table 1 — Clinical information of patients with cervical squamous cell carcinoma (CESC). [file Table_8.DOCX]

**Table S1 Clinical information of CESC patients**

| **Clinical variables** | **Number** | **Percentage (%)** |
| --- | --- | --- |
| Vital status |  |  |
| Alive | 236 | 76.873 |
| Dead | 71 | 23.127 |
| Age |  |  |
| <=65 | 272 | 88.599 |
| >65 | 35 | 11.401 |
| Tumor grade |  |  |
| G1-2 | 154 | 50.163 |
| G3-4 | 121 | 39.414 |
| unknown | 32 | 10.423 |
| AJCC-T |  |  |
| T1-2 | 213 | 69.381 |
| T3-4 | 31 | 10.098 |
| unknown | 63 | 20.521 |
| AJCC-M |  |  |
| M0 | 116 | 37.785 |
| M1 | 10 | 3.257 |
| unknown | 181 | 58.958 |
| AJCC-N |  |  |
| N0 | 135 | 43.974 |
| N1 | 60 | 19.544 |
| Unknown | 112 | 36.482 |
